# Nutcracker Syndrome Masquerading as Renal Colic in an Adolescent Athlete: A Case Report

**DOI:** 10.5811/cpcem.2021.6.52140

**Published:** 2021-08-27

**Authors:** Ron D. Waldrop, Paul Henning

**Affiliations:** * USA Health Systems, Department of Pediatric Emergency Medicine, Mobile, Alabama; † USA Health Systems, Department of Emergency Medicine, Mobile, Alabama

**Keywords:** renal vein, abdominal pain, flank pain, nutcracker syndrome, hematuria, proteinuria, case report

## Abstract

**Introduction:**

Abdominal pain and flank pain cause a significant proportion of emergency department (ED) visits. The diagnosis often remains unclear and is frequently associated with repeat visits to the ED for the same complaint. A rare cause of left upper abdominal and flank pain is compression of the left renal vein between the aorta and the superior mesenteric artery known as nutcracker syndrome. Diagnostic findings on ultrasound include increased left renal vein diameter proximal and peak blood flow velocity increase distal to the superior mesenteric artery. We describe such a patient presenting to an ED repeatedly with severe pain mimicking renal colic before the final diagnosis and intervention occurred.

**Case Report:**

A 16-year-old female, long-distance runner presented four times complaining of intractable left upper quadrant abdominal pain radiating to the left flank after exercise. On each visit urinalysis revealed proteinuria and hematuria, and on two visits abdominal computed tomography revealed no kidney stone or dilatation of the collecting system. Ultimately, she was referred to vascular surgery where Doppler ultrasonography was used to diagnose left renal vein compression. Transposition of the left renal vein improved Doppler diameter and flow measurements and eliminated symptoms.

**Conclusion:**

Emergency physicians must maintain a large list of possible diagnoses during the evaluation of abdominal and flank pain with a repetitive and uncertain etiology. Nutcracker syndrome may mimic other causes of abdominal and flank pain such as renal colic and requires appropriate referral.

## INTRODUCTION

Abdominal and flank pain are common complaints in patients presenting to the emergency department (ED).^1^ The workup is often extensive, and the etiology frequently remains unclear at the time of discharge. Patients are often given pain relief and sent home for observation and subsequent follow-up. Nonetheless, the repetitive nature of this pain, especially when the cause is uncertain, results in repeat visits to the ED. A rare cause of recurrent left upper abdominal and flank pain is compression of the left renal vein between the aorta and the superior mesenteric artery (SMA). When symptomatic it is known as nutcracker syndrome (NS).^2^,^3^ To our knowledge NS has not been addressed in the emergency medicine literature and may not be familiar to emergency physicians.

In this case report we describe an adolescent female athlete who presented on four different occasions to the ED with presumed intermittent renal colic due to kidney stones, associated with hematuria and proteinuria. After significant delay, she was ultimately diagnosed with NS and received curative surgical correction.

## CASE REPORT

The patient was a 16-year-old female, long-distance runner with a body mass index of 19 who complained of left upper quadrant abdominal pain radiating to the flank after exercise. She presented to the ED on four occasions over six months with normal vital signs and a complaint of severe pain that resolved with opiate pain management. Abdominal examination each time was not consistent with an emergent surgical problem requiring immediate consultation. Each time urinalysis showed proteinuria and hematuria. On two visits a non-contrast abdominal computed tomography (CT) was performed, which revealed no kidney stone, ureteral calculi, or dilatation of the collecting system.

On the first three occasions after pain management she received instructions to follow up with primary care for further evaluation. After the fourth episode the patient was referred to vascular surgery with a clinical suspicion of NS. Doppler ultrasonography in the standing position demonstrated compression of the left renal vein by the SMA with a hilar (proximal) left renal vein diameter of 9.12 millimeters (mm) and aortico-mesenteric (distal) diameter of 2.1 mm (ratio 4.34), as well as an aortico-mesenteric peak velocity of 141 centimeters per second (cm/sec) and a hilar peak velocity of 20.1 cm/sec (ratio 6.97). The patient ultimately underwent renal vein transposition to a lower aortic position with elimination of her symptoms and resumption of her running career.

## DISCUSSION

Abdominal and flank pain account for up to 10% of all ED visits.^1^ Diagnoses may range from mild and self-limiting to life-threatening disease. Because of the repetitive nature and overlapping clinical appearance of this pain as well as the frequently uncertain diagnosis, these patients are prone to diagnostic delay resulting in repeat visits to the ED until definitive referral, management, and intervention.

**Figure 1:**
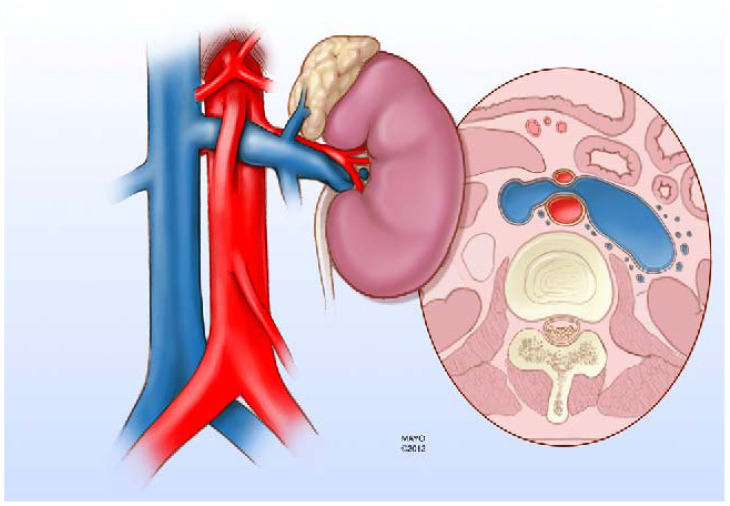
Illustration showing the anterior nutcracker syndrome in which the left renal vein is compressed as it passes between the aorta and the superior mesenteric artery resulting in dilation of the portion proximal to the kidney hilum. Reproduced from open access reference 4.

The SMA originates from the aorta behind the neck of the pancreas at the level of the first lumbar vertebra and creates an angle at its origin from the aorta known as the SMA angle or aorto-mesenteric angle.^4^ The left renal vein (LRV) and third part of the duodenum pass in this space leading to two uncommon syndromes: SMA syndrome with compression of the SMA, and anterior nutcracker syndrome (NS) with compression of the LRV when the angle is too acute or devoid of adipose^5^ (Figure 1).

Rarely, the LRV arises posterior to the aorta and compression of the LRV may occur between the aorta and vertebral body, which when symptomatic is known as posterior NS. Presence of LRV compression without symptoms is referred to as nutcracker phenomenon.^2^ Left renal vein compression in anterior NS impairs LRV blood outflow and is characterized by distention of the hilar (proximal to obstruction) portion of the vein and results in elevation of the peak velocity in the aortico-mesenteric (distal to obstruction) LRV.^6^ The most common embryologic explanation for NS is abnormally low or lateral origin of the SMA resulting in an angle of less than 90 degrees.^7^ In addition, an abnormally high course of the LRV may contribute to symptoms. Standing may also cause this angle to become more constricted leading to more LRV congestion.^8^

**CPC-EM Capsule**

What do we already know about this clinical entity?

*Nutcracker syndrome (NS) is caused by compression of the left renal vein by the superior mesenteric artery, causing episodic severe left flank pain.*

What makes this presentation of disease reportable?

*The patient had repeated clinical presentation to the Emergency Department with symptoms that mimicked renal colic despite lack of imaging evidence to support that diagnosis.*

What is the major learning point?

*A wider differential diagnosis that includes NS could expedite accurate diagnosis, especially in patients with negative imaging studies and repeated visits.*

How might this improve emergency medicine practice?

*Emergency physicians should consider a wider differential diagnosis for patients with left flank pain resembling renal coli.*

Symptoms resulting from LRV compression in NS were first described in 1950 and have been demonstrated in ages ranging from childhood to the seventh decade.^2^,^9^ Left renal vein compression is believed to cause LRV hypertension and stasis resulting in left-sided abdominal or flank pain with microscopic or macroscopic hematuria resulting from periureteral and gonadal varices, as well as proteinuria by either random or orthostatic sample in children.^10^ Hematuria appears to be due to ureteral bleeding as demonstrated on cystoscopy.^11^ Nutcracker syndrome is notoriously difficult to diagnose because it mimics many other clinical syndromes such as renal colic due to kidney stones. Some patients may report symptoms exacerbated by standing or walking. Finally, a variety of other symptoms including abdominal pain and neuroendocrine orthostasis have been described and are thought to represent an unknown, associated autonomic disturbance.^12^

Compression of the LRV resulting in NS may be demonstrated by venography, CT arteriography, Doppler ultrasonography (DUS), and magnetic resonance imaging.^13^ To decrease invasiveness and expense without sacrificing accuracy, DUS is the most used method to measure diameter and peak velocity in both the hilar and aorto-mesenteric areas of the LRV. Diagnostic measurements from previous studies have suggested a hilar to aortico-mesenteric diameter ratio greater than 4.7 may be predictive of NS with 100% sensitivity and 90% specificity.^14^ An aortico-mesenteric to hilar peak velocity ratio of the LRV greater than 7 may also be suggestive, but the range of normal values in children is wide. Finally, DUS measurements in the standing position may be more accurate in demonstrating the effect of a narrow SMA angle on the LRV.^8^

**Figure 2:**
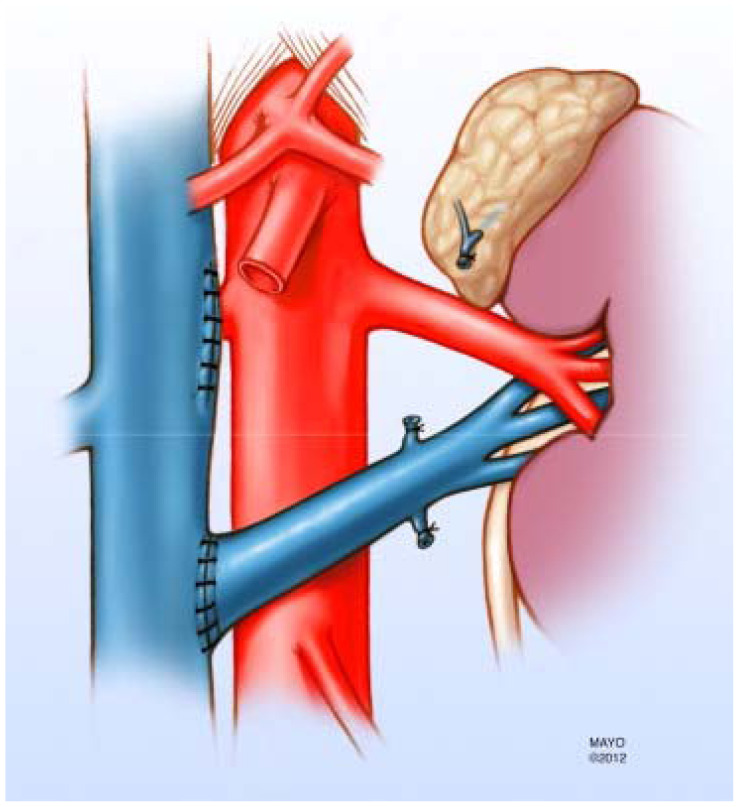
Illustration demonstrating representative surgical correction of compressed left renal vein in anterior Nutcracker syndrome. Reproduced from open access journal reference 4.

Nutcracker syndrome is associated with non-glomerular hematuria due to ureteral varices resulting from elevated LRV pressure.^10^ In case reports, NS has also been associated with co-existing Henoch-Schönlein purpura, immunoglobulin A (IgA) nephropathy, idiopathic hypercalciuria with nephrolithiasis, and membranous nephropathy. Due to the symptom overlap of NS and IgA nephropathy and glomerulonephritis with the presence of flank pain and hematuria, it has been suggested NS should be ruled out with appropriate imaging before diagnostic renal biopsy is performed.^15^ Treatment depends on severity and includes conservative and surgical approaches^4^,^9^ (Figure 2). Single or rare occurrences may resolve over time with body habitus change and subsequent deposition of fat along the mesentery preventing angle compression of the LRV. For intractable or recurrent NS, surgical correction may be warranted, with transposition of the LRV out of the SMA angle being the most common approach.

## CONCLUSION

Abdominal and flank pain are common complaints in patients presenting to the ED. Frequently, diagnostic testing is inconclusive resulting in an unclear diagnosis and repeat visits to the ED for the same complaint. In the face of this circumstance, emergency physicians must maintain a wide differential diagnosis and low threshold for referral to appropriate specialists. Nutcracker syndrome symptoms and signs may mimic other, more common causes of recurrent abdominal and flank pain such as renal colic due to kidney stones and should be considered by emergency physicians in the differential diagnosis of such patients when diagnostic studies are inconclusive.

## References

[1] Hijaz NM, Friesen CA (2017). Managing abdominal pain in pediatric patients: current perspectives. Pediatric Health Med Ther.

[2] Kurklinsky AK, Rooke TW (2010). Nutcracker phenomenon and nutcracker syndrome. Mayo Clin Proc.

[3] Pournasiri Z (2016). The nutcracker syndrome as a rare cause of chronic abdominal pain: a case report. J Compr Ped.

[4] Said M, Gloviczki P, Kalra M (2013). Renal nutcracker syndrome: surgical options. 2013. Semin Vasc Surg.

[5] Arthurs OJ, Mehta U, Set PAK (2012). Nutcracker and SMA syndromes: What is the normal SMA angle in children?. J Rad.

[6] Ozcakar Z, Birsin Y, Inkaya F (2011). Nutcracker syndrome manifesting with severe proteinuria: a challenging scenario in a single-kidney patient. Pediatric Nephrol.

[7] Hohenfellner M, Steinbach F, Schultz-Lampel D (1991). Nutcracker syndrome: new aspects of pathophysiology, diagnosis, and treatment. J Urol.

[8] Fitoz S, Ekim M, Ozcakar ZB (2007). Nutcracker syndrome in children: the role of upright position examination and superior mesenteric artery angle measurement in the diagnosis. 2007. J Ultrasound Medicine.

[9] Alaygut D, Bayram M, Soylu A (2013). Clinical course of children with nutcracker syndrome. Urology.

[10] Kavukcu S, Kasap B, Goktay Y (2004). Doppler sonographic indices in diagnosing the nutcracker phenomenon in a hematuric adolescent. J Clin Ultrasound.

[11] Park SJ, Lim JW, Cho BS (2002). Nutcracker syndrome in children with orthostatic proteinuria: diagnosis on the basis of Doppler sonography. J Ultrasound Med.

[12] Mazzoni MB, Kottanatu L, Simonetti GD (2011). Renal vein obstruction and orthostatic proteinuria: a review. Nephrol Dial Transplant.

[13] Miro I, Serrano A, Perez-Ardavin J (2020). Eighteen years of experience with pediatric nutcracker syndrome: the importance of the conservative approach 2020. J Ped Urology.

[14] Cheon JE, Kim WS, Kim IO (2006). Nutcracker syndrome in children with gross haematuria: Doppler sonographic evaluation of the left renal vein. Pediatric Radiology.

[15] Ma Z, Liu X, Ning Y (2013). Nutcracker phenomenon in combination with glomerular nephritis in isolated hematuria patients. Int Urol Nephrol.

